# Long-duration human spaceflight induces atrophy in the left ventricular papillary muscles

**DOI:** 10.1038/s41526-025-00531-7

**Published:** 2025-11-12

**Authors:** C. Tordeur, E. Abdessater, A. Hossein, F. Righetti, V. Sinitsyn, E. Mershina, E. Luchitskaya, E. G. Caiani, V. Faoro, J. Tank, P. van de Borne, P.-F. Migeotte, J. Rabineau

**Affiliations:** 1 Laboratory of Physics and Physiology (LPHYS), Department of Cardiology, Hôpital Universitaire de Bruxelles - Erasme Hospital, Université libre de Bruxelles, Brussels, Belgium; 2 Faculty of Medicine, Université libre de Bruxelles, Brussels, Belgium; 3 Research Unit in Cardio-Respiratory Physiology, Exercise & Nutrition, Faculty of Human Movement Sciences, Université libre de Bruxelles, Brussels, Belgium; 4 Brussels Laboratory of the Universe (BLU-ULB), Université libre de Bruxelles, Brussels, Belgium; 5 Electronics, Information and Bioengineering Dpt., Politecnico di Milano, Milan, Italy; 6 Department of Radiology, Medical Educational and Scientific Center University Hospital, Lomonosov Moscow State University, Moscow, Russia; 7 Institute of Biomedical Problems of the Russian Academy of Sciences, Moscow, Russia; 8 IRCCS Istituto Auxologico Italiano, San luca Hospital, Milan, Italy; 9 Institute of Aerospace Medicine, German Aerospace Center (DLR), Cologne, Germany; 10 Department of Kinesiology and Health Sciences, University of Waterloo, Waterloo, Canada

**Keywords:** Magnetic resonance imaging, Physiology, Anatomy

## Abstract

Microgravity exposure induces cardiac deconditioning, primarily due to hypovolemia and inactivity. Animal models suggest microgravity may cause left ventricular (LV) papillary muscle atrophy, but this has not been studied in humans. This study used MRI to assess LV papillary muscle mass and LV morphology and function in nine male cosmonauts before and 6 ± 2 days after long-duration spaceflight (247 ± 90 days). Spaceflight did not affect LV volumes, ejection fraction, and strain parameters, but increased heart rate (*P* < 0.001) and cardiac output (*P* = 0.03). LV papillary muscle mass decreased by 14% (*P* = 0.017), while LV mass tended to increase (*P* = 0.083), mitral annular diameter increased (*P* = 0.004) without mitral leakage, and LV sphericity increased (*P* = 0.02). These findings suggest LV adapts to space with geometric changes, but microgravity-induced papillary muscle atrophy requires further study for long-term implications.

## Introduction

Exposure to microgravity induces a cranial fluid shift^1^. This partial vascular redistribution leads to an initial atrial expansion^2^ and a decrease in total plasma volume^3^. Moreover, the removal of the downward pull of gravitational forces from the Earth causes a reduction in mechanical loading on the longitudinal axis of the heart^4,5^. Altogether, without exercise countermeasures, long-term exposure to such conditions leads to a decrease in left ventricular (LV) function, as assessed by transthoracic echocardiography, with a reduction in LV stroke volume (SV)^6^ and increased sphericity of the LV cavity^7^. Furthermore, after a few weeks in microgravity, apparent atrophy of the LV was observed using cardiac magnetic resonance imaging (MRI) in the absence of exercise countermeasure^8^.

Simulated microgravity through -6° head-down bed-rest (HDBR) is considered a valid Earth-based model of microgravity because it elicits most of the physiological effects of microgravity exposure through inactivity and a cranial fluid shift^9–11^. It induces a decrease in total plasma volume of ~6–15% after a few days to 45 days^11,12^. Cardiovascular deconditioning is also an induced effect of -6° HDBR without exercise countermeasures, with supporting evidence of an induced decrease in LV function^13–16^, a reduction in LV strain mechanics^17^, and a reduction in LV myocardial mass^18–20^.

However, no definitive explanation has been provided regarding the mechanisms underlying apparent LV atrophy. Dehydration caused by physiological fluid exchanges provoked by microgravity-induced fluid redistribution, instead of real cellular atrophy, could be the cause of this reduction in LV myocardial mass^21^. Indeed, a return to pre-flight LV myocardial mass values was observed soon after spaceflight using transthoracic echocardiography^21^. Additionally, the observed LV atrophy could be reproduced under ground-based dehydration condition^21^. The -6° HDBR model was used to test the effectiveness of countermeasures to prevent cardiovascular deconditioning, resulting in either preservation^18,20,22^ or an increase^19,23^ in the LV myocardial mass. Recently, a positive effect of exercise countermeasures on the preservation of LV myocardial mass was demonstrated after long-duration spaceflight onboard the International Space Station (ISS)^24^.

However, in these previous studies, no specific focus has been placed on the LV papillary muscles (PPM), and LV mass quantification was usually performed by pre- and post-flight measurements using cardiac MRI, considering the PPM as part of the LV cavity. The only study investigating modifications in PPM after exposure to microgravity was published by Goldstein et al.^25^ in 1992 in a murine animal model: after exposing rats to 14 days of microgravity during the COSMOS 2044 spaceflight, a decrease of 19% in the myofiber cross-sectional area of the PPM was observed compared with the ground control. However, no changes in the LV myofiber cross-sectional area were observed, probably because of the short duration of exposure, with no associated measurements of LV functional adaptation. Nevertheless, this study suggests that microgravity affects the PPM differently than the LV parietal myocardium. Anatomically, the PPM are two critical structures attaching postero-medially and antero-laterally to the ventricular myocardium and connected to the mitral valve (MV) cusps via the chordae tendineae^26^. The primary role of the PPM is to prevent the inversion or prolapse of the MV leaflets during systole by contracting and maintaining tension on the chordae tendineae, thereby ensuring proper MV closure and unidirectional atrioventricular blood flow. PPMs are therefore essential for the proper functioning of the MV and thus contribute significantly to the LV work^26–28^.

In clinical practice, quantification of LV myocardial mass using cardiac MRI has previously been reported both including and excluding the PPM in the LV mass computation^29–31^. This choice has significant implications, as their inclusion may hinder PPM changes, especially in studies examining the effects of microgravity on the cardiovascular system where small changes in total LV mass are expected. The PPM accounts for ~9% of the total LV myocardial mass^29^, which corresponds to a value comparable to the total LV atrophy reported following microgravity exposure^21^. Thus, excluding PPM from LV myocardial mass quantification is essential for isolating and accurately assessing specific changes in LV mass and PPM morphology.

Given the paucity of literature on this subject, there is a significant need to evaluate the impact of potential alterations in PPM in humans exposed to microgravity and to include functional measurements, especially in the context of extended exposure to microgravity. Accordingly, our aim was to use cardiac MRI as a valuable and appropriate imaging modality to assess and quantify changes in the PPM and ventricular myocardial mass^32^ induced by extended exposure to microgravity in cosmonauts. We hypothesized that the mass of the PPM would decrease in cosmonauts after long-term spaceflight, with concomitant modifications in the LV morphology and function, as well as changes in the structure of the MV.

## Results

### Reproducibility assessment

The intra-rater reliability and inter-rater agreement results of the analyses conducted with the intraclass correlation coefficients (ICC) were all between good and excellent (as reported in Tables 1, 2, and 3 for the intra-rater and inter-rater analysis). No biases were identified by Bland-Altman and the statistical relationships were the same for all iterations of measurements.

**Table 1:** Left ventricular function

Parameters		Spaceflight timepoints		*P* value	ICCs	
		PRE	POST		Intra	Inter
		(*n* = 9)	(*n* = 9)			
EDV	(mL)	164.8 ± 24.6	162.6 ± 27.0	0.459	0.97	0.95
ESV	(mL)	62.3 ± 16.6	61.3 ± 16.9	0.844	0.89	0.87
SV	(mL)	102.5 ± 21.2	101.4 ± 16.8	0.792	0.89	0.88
EF	(%)	62.1 ± 8.3	62.5 ± 6.2	0.890	0.89	0.78
HR*	(bpm)	51 ± 7	59 ± 6	**< 0.001**	NA	NA
CO	(L/min)	5.2 ± 1.3	6.0 ± 1.0	**0.030**	0.94	0.93
GLS	(%)	−16.15 ± 1.79	−15.89 ± 1.89	0.645	0.96	0.96
GCS	(%)	−18.23 ± 2.47	−17.59 ± 2.35	0.432	0.97	0.97

*PRE* pre-flight, *POST* post-flight, *ICC* intraclass correlation coefficient, *EDV* end-diastolic volume, *ESV* end-systolic volume, *SV* stroke volume, *EF* ejection fraction, *HR* heart rate, *CO* cardiac output, *GLS* global longitudinal strain, *GCS* global circumferential strain, *NA* not applicable. *P* values reported in bold are considered statistically significant. * Heart rate was measured at the bedside of the cosmonauts just before the MRI acquisitions.

**Table 2:** Left ventricular morphology and mitral-valve-related parameters

Parameters		Spaceflight timepoints		*P* value	ICCs	
		PRE	POST		Intra	Inter
		(*n* = 9)	(*n* = 9)			
Myocardial mass	(g)	137.3 ± 23.5	150.6 ± 30.1	*0.083*	0.97	0.89
Diameter	(mm)	49.5 ± 4.1	49.5 ± 4.5	0.904	0.97	0.96
Length	(mm)	101.3 ± 8.2	99.2 ± 8.1	**0.020**	0.99	0.98
Sphericity index	(−)	0.49 ± 0.05	0.50 ± 0.05	**0.018**	0.98	0.96
LVEDd	(mm)	53.5 ± 3.4	52.4 ± 3.3	*0.067*	0.99	0.95
IVSd	(mm)	9.6 ± 1.5	10.5 ± 1.9	**0.031**	0.97	0.95
LVPWd	(mm)	6.8 ± 1.2	7.9 ± 1.1	**0.004**	0.95	0.92
RWT	(−)	0.31 ± 0.05	0.35 ± 0.05	**0.003**	0.96	0.94
PPM mass	(g)	10.1 ± 1.8	8.7 ± 1.8	**0.017**	0.97	0.84
PPM/LV mass ratio	(−)	0.075 ± 0.015	0.059 ± 0.009	**0.005**	0.98	0.81
MAD 2CV	(mm)	40.0 ± 3.9	38.8 ± 4.1	0.187	0.90	0.86
MAD 4CV	(mm)	34.8 ± 4.0	36.8 ± 4.4	**0.004**	0.97	0.91

*PRE* pre-flight, *POST* post-flight, *ICC* intraclass correlation coefficient, *LVEDd* left ventricular end-diastolic diameter, *IVSd* left ventricular septal wall thickness, *LVPWd* left ventricular posterior wall thickness, *RWT* relative wall thickness, *PPM* papillary muscles, *LV* left ventricle, *MAD* mitral annular diameter, *2CV* two-chamber long-axis view, *4CV* four-chamber long-axis view. *P* values reported in bold are considered statistically significant, and the ones reported in italic are considered as exposing a possible statistical trend.

**Table 3:** Left ventricular tissue properties

Parameters			Spaceflight timepoints		*P* value	ICCs	
			PRE	POST		Intra	Inter
			(*n* = 7)	(*n* = 7)			
T1 map	1.5 T	(ms)	961.4 ± 18.7	965.4 ± 28.4	0.297^$^	0.99	0.99
	3 T	(ms)	1144.8 ± 18.6	1202.8 ± 24.1			
T2 map	1.5 T	(ms)	42.5 ± 1.4	43.6 ± 1.3	*0.078*^$^	0.97	0.87
	3 T	(ms)	40.1 ± 0.4	40.6 ± 0.4			

*PRE* pre-flight, *POST* post-flight, *ICC* Intraclass correlation coefficient, T1 map left ventricular time constant for recovery of longitudinal magnetization, T2 map left ventricular time constant for the decay of transverse magnetization. *P* values reported in italic are considered as exposing a possible statistical trend. ^$^ Analysis on pooled 1.5 T (*n* = 5) and 3 T (*n* = 2) samples.

### Changes in LV function

Table 1 lists the results related to the LV function. No significant differences in end-diastolic volume (EDV), end-systolic volume (ESV), SV, and ejection fraction (EF) were observed between pre- and post-flight. However, due to a higher heart rate (HR) post-flight compared with pre-flight (59 ± 6 vs. 51 ± 7 bpm; *P* < 0.001; *d* = 2.60), increased cardiac output (CO) was observed post-flight (6.0 ± 1.0 vs. 5.2 ± 1.3 L/min; *P* = 0.030; *d* = 0.88). Regarding the LV strain results, global longitudinal strain (GLS) and global circumferential strain (GCS) did not change.

### Changes in LV morphology and mitral-valve-related parameters

**Fig. 1:**
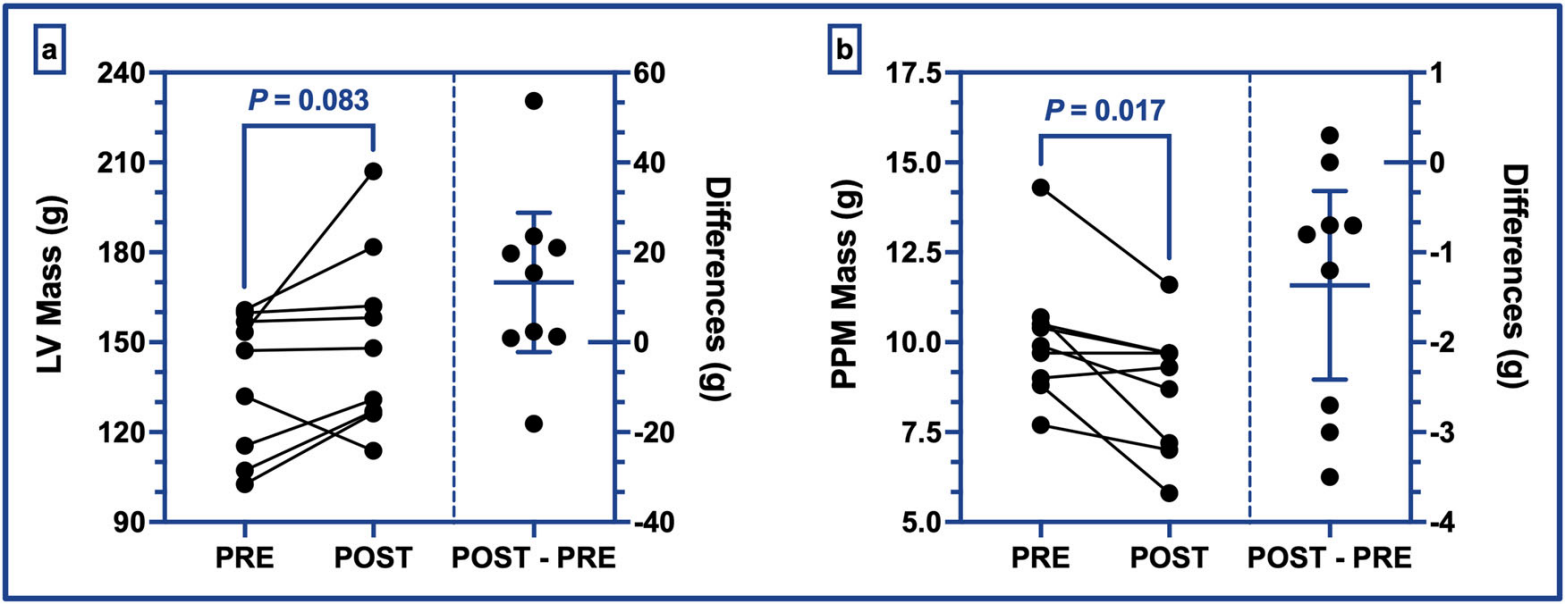
Left ventricular and papillary muscle masses. **a** represents the evolution of the left ventricular myocardial mass quantified before and after spaceflight (left axis). **b** represents the evolution of the left ventricular papillary muscles mass quantified before and after spaceflight (left axis). Each right subpanel represents individual POST–PRE differences in addition to the mean difference between the two groups with its 95% confidence interval (right axis). Individual values are plotted in overlay (black circles). *P* value < 0.05 is considered statistically significant, and *P* value < 0.1 is considered to uncover possible trends. PRE pre-flight, POST post-flight, POST–PRE individual POST–PRE difference, LV left ventricle, PPM papillary muscles.

**Fig. 2:**
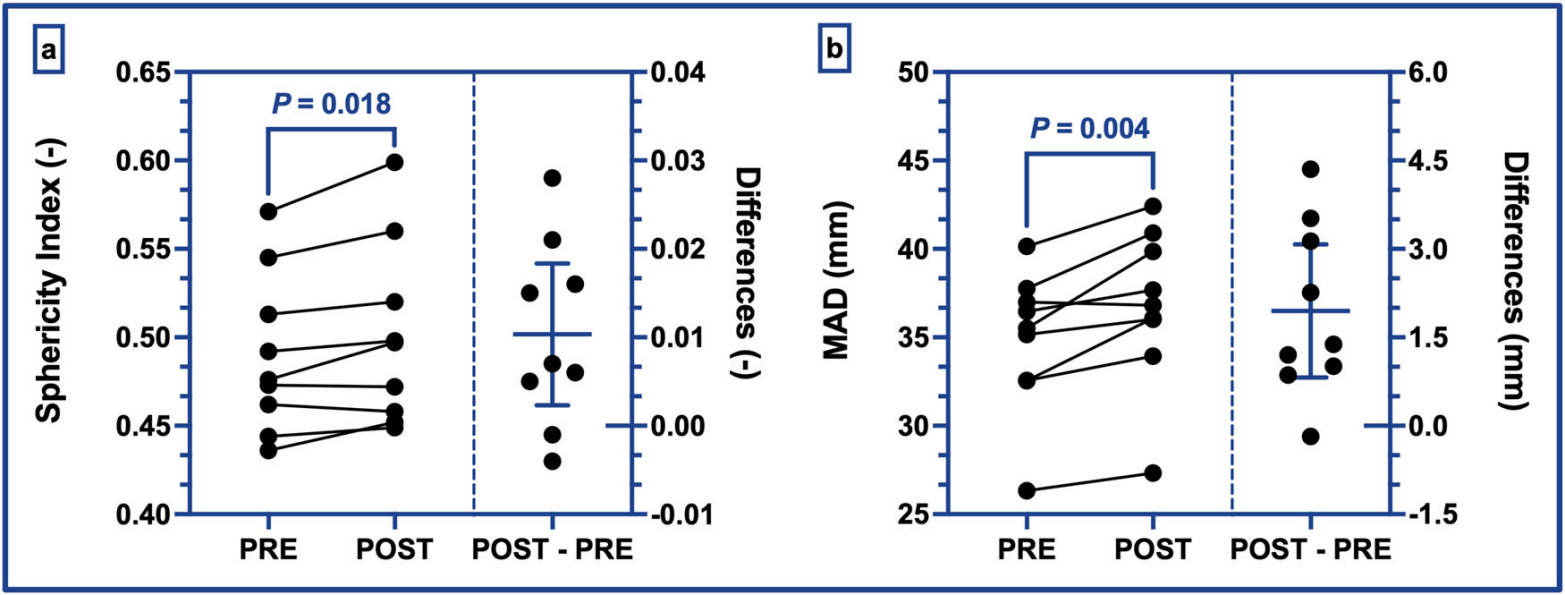
Left ventricular structural changes. **a** represents the evolution of the left ventricular sphericity index quantified before and after spaceflight (left axis). **b** represents the evolution of the mitral annular diameter quantified before and after spaceflight (left axis). Each right subpanel represents individual POST—PRE differences in addition to the mean difference between the two groups with its 95% confidence interval (right axis). Individual values are plotted in overlay (black circles). *P* value < 0.05 is considered statistically significant. PRE pre-flight, POST post-flight, POST–PRE individual POST–PRE difference, MAD mitral annular diameter measured in four-chamber long-axis view in end-diastole.

Table 2 lists the results related to LV morphology. No changes were observed in the LV diameter, but a possible trend towards an increase in LV myocardial mass post-flight (150.6 ± 30.1 vs. 137.3 ± 23.5 g; *P* = 0.083; *d* = 0.66; see Fig. 1a) was observed. Moreover, a decrease in LV length (99.2 ± 8.1 vs. 101.3 ± 8.2 mm; *P* = 0.020; *d* = 0.97) and an increase in the LV sphericity index (SI, 0.50 ± 0.05 vs. 0.49 ± 0.05; *P* = 0.020; *d* = 0.99; see Fig. 2a) were observed post-flight compared with pre-flight values. These post-flight geometric changes were accompanied by an increase in relative wall thickness (RWT, 0.35 ± 0.05 vs. 0.31 ± 0.05; *P* = 0.003; *d* = 1.39), driven by increases in both LV posterior wall thickness (LVPWd, 7.9 ± 1.1 vs. 6.8 ± 1.2 mm; *P* = 0.004; *d* = 1.33) and LV septal wall thickness (IVSd, 10.5 ± 1.9 vs. 9.6 ± 1.5 mm; *P* = 0.031; *d* = 0.87), while LV end-diastolic diameter tended to decrease (LVEDd, 52.4 ± 3.3 vs. 53.5 ± 3.4 mm; *P* = 0.067; *d* = −0.71). In addition, a 14% decrease (relative difference between the average of the pre-flight and the average of the post-flight values) in PPM mass (8.7 ± 1.8 vs. 10.1 ± 1.8 g; *P* = 0.017; *d* = −1.0; see Fig. 1b) was observed compared with pre-flight values (mean of differences with 95% CI = −1.36 g [−2.42 to −0.32 g]). In line with these changes, the ratio of PPM/LV mass showed a decrease post-flight (0.059 ± 0.009 vs. 0.075 ± 0.015; *P* = 0.005; *d* = −1.26). No changes were found in the mitral annular diameter (MAD) measured in the LV two-chamber (2CV) view, whereas a significant increase in MAD in the LV four-chamber (4CV) view (36.8 ± 4.4 vs. 34.8 ± 4.0 mm; *P* = 0.004; *d* = 1.33; see Fig. 2b) was observed compared with pre-flight.

### Changes in LV tissue properties

Table 3 presents the results of the LV tissue mapping. No differences were observed in the pooled time constant of longitudinal magnetization recovery without an exogenous contrast agent (native T1). However, a possible trend towards an increase in the time constant of the decay of transverse magnetization (T2) was observed post-flight compared to pre-flight values.

### Correlations

No correlations were found between changes in PPM mass and mitral annular diameter, LV myocardial mass, LV SI, LV length, and LV strain parameters, GLS, and GCS. No correlations were found between strain parameters and HR, SV, CO, LV myocardial mass, and LV SI. Additionally, a trend towards a positive correlation between changes in native T1 and LV myocardial mass was found (*R* = 0.74, *R*^2^ = 0.54, F(1, 5) = 5.943, *P* = 0.059) with a fitted linear regression model as follows: ΔLV mass = 2.820*Δnative T1 + 3.763.

## Discussion

This study evaluated the effects of long-term exposure to microgravity on cardiac LV morphology and function using MRI. The main new finding is that spaceflight induces a complex pattern of cardiac LV remodeling, characterized by a significant decrease in PPM mass alongside a trend towards increased LV myocardial mass, as well as an increase in the MV annular diameter. Notably, these structural changes occurred in the absence of significant variations in LV volumes (EDV and ESV), suggesting that microgravity exposure induces geometric remodeling, rather than volumetric changes. Supporting this hypothesis, an increased LV sphericity index was observed due to a decrease in the LV longitudinal length from pre-flight to post-flight. Furthermore, a post-flight decrease in the PPM-to-LV mass ratio was observed, indicating that the myocardium of the PPM and the LV parietal myocardium adapt differently to microgravity. These findings suggest regional myocardial adaptations to prolonged unloading in microgravity, where different components of the LV myocardium may undergo divergent remodeling trajectories.

This study is the first to investigate the effects of long-duration spaceflight on PPM in humans. The 14% reduction in PPM mass post-flight represents a novel observation in the context of human cardiac adaptation to long-duration microgravity. Notably, the PPM-to-LV mass ratio significantly decreased after spaceflight, emphasizing the selective remodeling response in PPM compared to global LV myocardial mass. Looking at animal models, a study investigating a two-week microgravity exposure in rats has previously documented a 19% decrease in the myofiber cross-sectional area of the PPM compared with ground control rats^25^. Additionally, simulating microgravity in rodents through a 4-week tail suspension also showed impaired contractile function in PPM myocardium, with decreased tension force, maximal velocity of contraction, and a decrease of 19% in the myocardial myofibrillar Ca^2+^-ATPase activity compared with the control group^33,34^.

From a physiological standpoint, the PPM display unique characteristics compared to the surrounding LV myocardium. Unlike the latter, they exhibit a distinct electromechanical behavior, characterized by delayed electrical activation relative to the LV free wall and a contraction profile that begins during the ejection phase, continues throughout systole, and extends into isovolumic relaxation. This unique timing supports the chordal tension required for mitral-valve competence^35–37^. In contrast, PPM lengthening occurs during late diastole and early systole, exposing them to repetitive mechanical loading and unloading. The PPM also experience peak tension while being elongated during systole and reach their lowest tension during early diastole when they are fully contracted and shortened. Additionally, it is also important to highlight that the PPM might have a heightened susceptibility to atrophy in chronic unloading conditions like microgravity compared to LV parietal myocardium, as PPM cardiomyocytes have a more longitudinal orientation that may amplify mechanical uncoupling as gravitational unloading disrupts axial strain patterns^38^.

Altered mechanical loading across the ventricular wall may decrease transmitral stress and affect the PPM contractile function, particularly given their concentric contraction that extends into isovolumic relaxation, thereby amplifying their selective vulnerability under unloading conditions.

In contrast, the parietal myocardium may exhibit an opposite response, such as compensatory hypertrophy when exercise countermeasures are applied. Consistent with this region-specific remodeling, the absence of significant changes in EDV and ESV in our study, despite the reduction in PPM mass and the observed geometric alterations, suggests that shape, rather than volume, is the primary driver of PPM adaptation under long-duration spaceflight with exercise countermeasures. The increase in the LV SI, primarily due to a reduction in LV longitudinal length, is consistent with previous reports of spherical remodeling during microgravity exposure in humans^4,7^, and also underscores the role of gravitational load in shaping LV geometry, as evidenced by comparisons between standing and supine positions in humans^39^.

From a mechanistic perspective, a global reduction in mechanical load on the PPM through spherical remodeling of the LV and changes in force transmission in the structural complex, generated by the interaction between the MV and LV^40,41^, could lead to lower stimulation of local mechanoreceptors in the muscle tissue. This would result in decreased synthesis of contractile proteins and, consequently, muscle atrophy. This atrophy of the PPM could have a direct impact on the contractile properties of the muscle, with possible associated alterations in the excitation-contraction coupling mechanisms. Both mechanisms may contribute to a decreased maximal contraction velocity and developed tension force of the PPM. Further work is needed to address the functional implications of exercise countermeasures in this context, given the importance of the PPM in the normal function of the MV.

The PPM atrophy may be viewed as a localized adaptation to altered LV geometry and load rather than systemic myocardial loss. Specific research protocols should deepen the investigation of the mechanisms behind this localized adaptation. They should address the cellular mechanisms underlying this differential plasticity response when exposed to the same stimuli. Advancing this understanding is essential to determine whether this response is adaptive or maladaptive and to develop potential strategies to mitigate its consequences. Undoubtedly, alterations of the PPM are known to be one of the clinical starting points of secondary mitral-valve prolapse^42^, which is the most common cause of primary moderate to severe mitral regurgitation in resource-abundant countries^43,44^.

In the present study, MV annular diameter, measured in the long-axis 4CV view, was increased after long-duration exposure to microgravity, which complements the findings of PPM atrophy. Although the same was not observed on the long-axis 2CV view, this constitutes a novel finding regarding the morphology of the MV in the context of long-duration spaceflight, as no other studies have investigated MV dilation after microgravity exposure. Although we could not assess MV prolapse and leakage directly, clinical literature has highlighted the association between MV annular dilation and MV prolapse or regurgitation^45–47^. Most importantly, in the context of the present study findings, it is crucial to highlight the work of Izumi et al. and Nordblom et al., who demonstrated that the size and position of the PPM could be implicated in the underlying mechanisms of functional MV regurgitation^48,49^.

Further studies using tailored imaging protocols are warranted to explore how these changes in MV annular and PPM geometry might predispose to subclinical MV insufficiency in microgravity or upon return to gravity environments. Additionally, more attention should be given to MV function in the long term after long-duration spaceflight missions, as MV regurgitation remains asymptomatic for prolonged periods and, before symptoms occur, irreversible damage can take place^50,51^. Reassuringly, to our current knowledge, none of these clinical manifestations were reported among astronaut crews after spaceflight.

The differential remodeling observed, PPM mass loss versus preserved LV mass, raises the question of the role played by systemic influences such as exercise countermeasures, hydration status, and body mass. Due to material and practical constraints of conducting in-flight astronaut studies, many investigations have focused on simulated microgravity using -6° HDBR, with or without exercise countermeasures, to assess LV function and morphology. Although relatively few studies employed MRI, those that did, across durations of 18, 21, and 70 days, showed that exercise countermeasures can preserve or even increase LV myocardial mass compared to non-exercising controls^18–20,22,23^. These interventions included continuous and high-intensity interval training, as well as resistance exercise. Some of these studies also demonstrated that exercise alone can prevent decreases in LV EDV through training-induced plasma volume expansion^18–20^; however, this prevention of LV EDV decrease was not consistently observed in all protocols^22,23^.

Beyond exercise, Shibata et al. showed that intravenous fluid infusion post-HDBR restored LV EDV^23^, suggesting plasma volume expansion could be used on an individual basis. In actual spaceflight, such strategies are mirrored by in-flight per os water-salt supplementation and post-flight intravenous infusion, both known to help restore plasma volume upon landing.

Only a limited number of studies have examined the direct effects of microgravity on LV function and structure. Perhonen et al. reported a trend towards decreased LV myocardial mass after short-duration spaceflight without countermeasures using cardiac MRI^8^, while Summers et al. confirmed reductions in both LV mass and EDV under similar conditions using echocardiography^21^. Notably, the inclusion by Summers et al. of a ground-based dehydration model reproduced these decreases, suggesting that plasma volume loss, rather than intrinsic cardiac atrophy, may underlie these structural changes. Conversely, in long-duration missions where exercise countermeasures were applied, Shibata et al. observed no significant changes in LV mass or volume^24^, indicating that such interventions can effectively offset cardiovascular deconditioning in space.

The present results are consistent with this literature. No alterations were observed in LV myocardial mass and volumes after long-duration spaceflight with exercise countermeasures. The trend towards increased LV mass may be related to the tailored in-flight exercise protocols used during these missions, as also noted by Shibata et al.^24^.

In this context, LV tissue mapping provides further insight. Although no significant changes were observed in native T1, we noted a trend towards increased T2 post-flight. This suggests that microgravity and its associated fluid shifts may influence myocardial tissue hydration and extracellular matrix properties, aligning with previous findings that link plasma volume changes to alterations in myocardial composition^52^. An increase in T2 may reflect changes in myocardial compliance or stiffness, possibly driven by interstitial fluid redistribution or subclinical alterations in the extracellular environment^53,54^.

Furthermore, we observed a trend towards a positive correlation between LV myocardial mass and septal native T1. This pattern has been described in pathological conditions such as hypertrophic cardiomyopathy and cardiac allograft rejection, where increases in LV mass due to hypertrophy, infiltration, or diffuse fibrosis are associated with elevated septal native T1 values^55,56^. In our study, the observed trend towards LV hypertrophic remodeling, evidenced by increased mass and RWT, may be biologically linked to early tissue changes reflected in native T1.

The preservation of longitudinal and circumferential strain suggests intact ventricular function, even in the presence of geometric remodeling. These results align with studies using the -6° HDBR model combined with exercise countermeasures, which showed preserved longitudinal and circumferential strain parameters^18–20,22^, supporting the growing body of evidence that exercise countermeasures during microgravity or simulation of microgravity help maintain LV strain and function.

Together, these structural, functional, and tissue-level findings emphasize the value of a multi-parametric approach for assessing cardiac adaptation to spaceflight. Our study, alongside the work of Shibata et al^24^, reinforces the importance of tailored exercise and hydration protocols to maintain cardiovascular performance in microgravity, and highlights the need for continued monitoring of myocardial tissue properties for long-term astronaut health.

To fully contextualize our findings and guide future investigations, it is important to acknowledge several limitations inherent to the present study. Post-flight MRI measurements were acquired on average 6 days after landing. Considering the rapid cardiovascular and hemodynamic recovery following return to Earth’s gravitational field^57^, it is possible that the amplitudes of the reported changes would have been larger or that some trends could have become significant if the measurements had been conducted earlier after landing. However, it is not possible to give a final status on the kinetics of reversibility based on the present study.

Notably, the total number of subjects recruited in this study is relatively low, which is a common fact in microgravity studies, due to obvious recruitment and logistical constraints. However, our results could have significant importance considering the small number of studies investigating cardiac adaptations to actual microgravity exposure during long-duration spaceflight and the growing interest in spaceflight becoming accessible to a more diverse population.

It is important to acknowledge the potential variability in individual adherence to the prescribed exercise countermeasure regimen. Not all cosmonauts may have followed the protocol as strictly as intended^58^, which could have contributed to inter-individual differences in physiological outcomes and thus represents a limitation in the generalizability of our findings. Furthermore, the use of in-flight per os water-salt supplements and the likely immediate post-flight intravenous infusion used for plasma volume expansion may have influenced the observed results if applied inconsistently across participants.

It is important to note that the present cardiac MRI protocols were not specifically designed to evaluate the MV morphology and function. Indeed, other specific MRI acquisition protocols allow for a thorough assessment of the MV morphology and function, including detecting MV prolapse and regurgitation if present^59^.

Lastly, changes in arterial blood pressure could have influenced the results. Unfortunately, arterial blood pressure was not measured simultaneously with the MRI protocols. However, other procedures conducted on the same cosmonauts measured arterial blood pressure at different timepoints relative to the MRI sessions (from -28 to +20 days relative to the pre-flight MRI session, and from the same day to +2 days relative to the post-flight MRI session). These measurements did not reveal any significant pre- vs. post-flight differences in systolic or diastolic blood pressure (117 ± 7 vs. 122 ± 5 mmHg, *P* = 0.891 and 78 ± 6 vs. 77 ± 7 mmHg, *P* = 0.935, respectively).

In conclusion, our study demonstrates that a 6-month or longer exposure to microgravity on the ISS, associated with exercise countermeasure, induces a reduction of the LV PPM mass but not of the LV mass. Concomitant with mitral annular dilation, this atrophy could lead to subclinical alterations of the MV function, particularly when the normal terrestrial gravity is restored or landing on another celestial body subject to a partial gravitational field compared to the terrestrial one. However, because of the limitations of the present MRI protocols, conclusions regarding MV function could not be fully investigated. Despite the PPM atrophy, the LV seems to adapt to microgravity-induced physiological adaptation in geometry, maintaining EDV, ESV, SV, and EF.

Future research with larger cohorts is needed to elucidate the causes and consequences of the microgravity-induced LV PPM atrophy and mitral annular dilation, as well as their potential to cause functional MV alterations post-flight. Dedicated MRI protocols and longitudinal follow-up will be essential to assess the persistence or recovery of these structural changes over time.

## Methods

### Study design

Professional cosmonauts assigned to 6-month and longer (long-duration) ISS spaceflights were eligible to participate in this before-and-after spaceflight investigation. This study was performed in accordance with the Declaration of Helsinki and approved by the Erasme University Hospital Ethics Committee (ref. P2017/332/CCBB406201732664), by the Biomedical Ethics Committee of the Institute of Biomedical Problems of the Russian Academy of Sciences from the 20th of June 2018 (ref. 474, Cardiovector 2–3), as well as by the medical board of the Human Research Multilateral Review Board (ref. 18-006, 18-001-Ren-1, 18-001-Ren-2). The cosmonauts were prospectively recruited on a voluntary basis after providing written informed consent (Multinational Space Station Human Research Informed Consent - Form 1418). Cosmonauts flying through missions other than Soyuz missions were excluded due to incompatibility with the logistics of the post-flight cardiac imaging protocol.

### Population definition

In total, nine male cosmonauts exposed to microgravity during long-duration missions onboard ISS (Expedition 63–69, spanning between early 2020 and late 2023) were studied. Among these cosmonauts (mean age: 44 ± 6 y, body height: 1.77 ± 0.05 m, body weight: 82 ± 8 kg, BMI: 26.3 ± 1.8 kg/m^2^), three participated in a 12-month mission (range: 355–371 days), while six were assigned to a 6-month mission (range: 176–194 days). These two subgroups were pooled in the context of this study.

### Protocol

MRI acquisitions were performed using 1.5 T or 3.0 T MRI scanner (Magnetom Aero or Magnetom Vida, Siemens, Erlangen, Germany) at the University Hospital of the Moscow State University. HR was measured at the bedside of the cosmonauts just before MRI acquisition. The cosmonauts were supine during the measurements, and no contrast agents were used. The total MRI time was 60 min. The overall MRI procedure was repeated before (60–45 days before launch) and after (6 ± 2 days after landing) spaceflight.

During the ISS spaceflight, the cosmonauts followed a strict countermeasure protocol to prevent and counteract the negative effects of weightlessness on cardiovascular and musculoskeletal systems^58^. The protocol implemented involved ~2.5 hours of physical activity per day, divided into two sessions and utilizing all available exercise devices onboard (cyclo-ergometer, treadmill, and advanced resistive exercise device). Training followed a repeating 4-day microcycle composed of three consecutive days of physical activity and one day dedicated to recovery^58^. Treadmill sessions were structured with variable intensity across the microcycle: high-intensity intervals on day 1, moderate intensity on day 2, and low intensity on day three. Approximately a month before the end of the space mission, cosmonauts transitioned to a prioritized locomotor activity program in addition to lower-body negative suits used as physical methods to produce an Earth-like fluid distribution, as well as per os water-salt supplements used to prevent fluid loss and maintain tolerance to gravitational overload during return to the Earth.

### Cardiac MRI acquisitions

Conventional retrospective electrocardiography-gated multi-breath-hold balanced steady-state free precession (bSSFP) cine sequences were selected. The scanning protocol, following international guidelines, included several cine sequences: 2CV, 3CV aligned with the center of the LV outflow tract, 4CV, and SAX^60^. The scanning range of the short-axis was adjusted to cover the entire LV from the base to the apex during diastole and systole. Cine MRI parameters used for the acquisitions are presented in Table 4.

**Table 4:** MRI parameters set for the cine acquisition protocols

Parameters		Left ventricular 2CV, left ventricular 3CV, and left ventricular 4CV	Left ventricular SAX stack
Repetition time	(ms)	36.4	42.4
Echo time	(ms)	1.16	1.11
Flip angle	(°)	55	54
Field-of-view	(mm^2^)	243 × 300	276 × 340
Spatial resolution	(mm^2^)	1.55 × 1.55	1.77 × 1.77
Cine frames per slice	(–)	25	25
Slice thickness	(mm)	6	8
Interslice gap	(mm)		2

*2CV* left ventricular two-chamber view, *3CV* left ventricular three-chamber view, *4CV* left ventricular four-chamber view, *SAX* left ventricular short-axis.

Furthermore, the myocardial tissue was characterized using advanced tissue mapping sequences in three slices (basal, mid-ventricular, and apical) of the LV. Two specific magnetic tissue properties were quantified to compute a parametric mapping: native T1 and T2. To acquire native T1, a modified look-locker inversion (MOLLI) recovery imaging protocol was used in the diastolic phase. A motion correction algorithm was used for the two mapping acquisitions^53,60^. The MRI tissue mapping parameters used for acquisition are presented in Table 5.

**Table 5:** MRI parameters set for the tissue mapping acquisition protocols

Parameters		Native T1	T2
Sequence	(–)	MOLLI	T2-prepared bSSFP
Echo times	(ms)	1.15	1.35
Repetition time	(ms)	283.08	219.14
Preparation pulses	(ms)		0/25/55
Flip angle	(°)	35	12
Number of sets	(−)	8	3
Imaging plane	(−)	SAX	SAX
Number of slices acquired	(−)	3	3
Slice thickness	(mm)	8	6
Interslice gap	(mm)	20	15
Spatial resolution	(mm^2^)	1.33 × 1.33	1.77 × 1.77
Field-of-view	(mm^2^)	289 × 340	290 × 340

*SAX* left ventricular short-axis, *MOLLI* modified look-locker inversion recovery, *bSSFP* balanced steady-state free precession.

### Data collection, reproducibility, and analysis

Data collection was performed in Moscow and then transmitted to Brussels for analysis. Data analysis was conducted using CAAS MR Solutions version 5.1.3. (Pie Medical Imaging, Maastricht, The Netherlands). The data generated by this software were then saved in tabular format for statistical analysis. Data handling was designed to ensure that it was impossible to distinguish between pre- and post-flight recordings: all data from all subjects were mixed and analyzed in a fully blinded and randomized manner, with no regard to identity or sequence order. The intra-rater reliability and inter-rater agreement were also assessed by performing the measurements independently by two investigators located in Brussels. The first investigator blindly repeated the procedure with 2 months in between the two measurements.

**Fig. 3:**
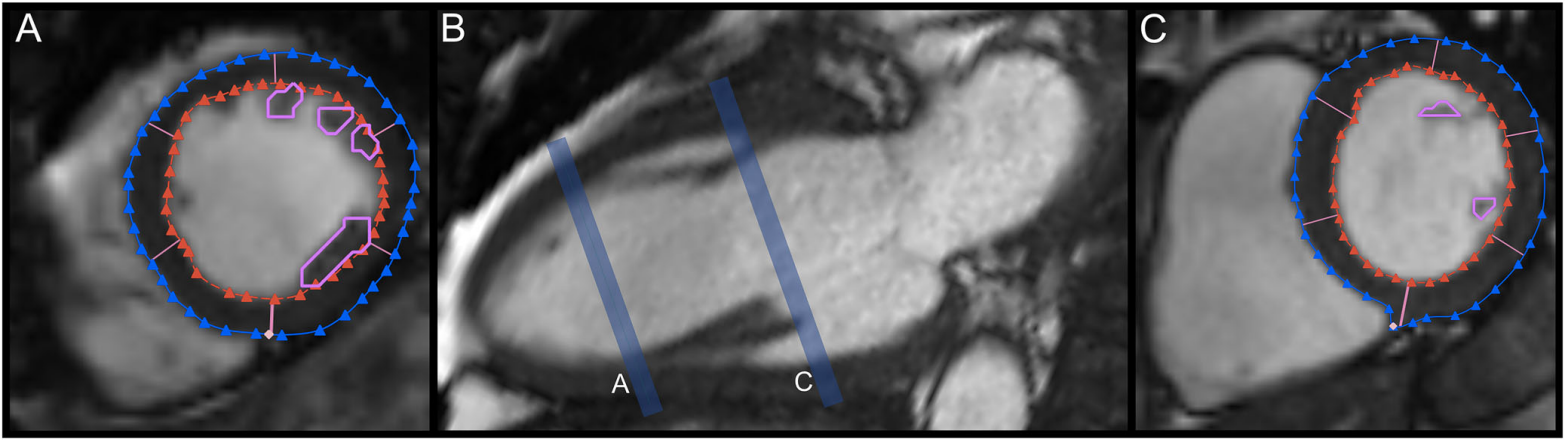
Visual representation of the slice considered as limits for the segmentation of the left ventricular papillary muscles. **A**, **C** are short-axis cine views of the left ventricle, and **B** is a long-axis cine view of the left ventricle. Slices represented in (**A**, **C**) intersect the long-axis plane in **B** at the corresponding letters to represent the slice considered as apical (**A**) and basal (**C**) limits for the segmentation of the left ventricular papillary muscles.

For each SAX acquisition, the end-diastolic (ED) and end-systolic (ES) frames, as well as the basal and apical planes, were defined according to the cardiovascular magnetic resonance guidelines^61^. The LV epicardium and endocardium were first segmented using an automatic segmentation tool applied to the images, followed by manual correction using a spline tool. Furthermore, a manual drawing of the contours of the LV PPM was performed. A standard of practice was defined to exclude LV trabeculae carnae and chordae tendineae from the PPM segmentation. Contouring was conducted on myocardial tissue with a concentric displacement during contraction, based on the following specific standard: PPM were segmented from the tip (slice C, Fig. 3B) to the root (slice A, Fig. 3B). No segmentation was performed on non-PPM cavitary myocardium, outside of their physiological anatomical locations (antero-lateral and postero-medial walls). Additionally, the segmentation was conducted on PPM taken together as the anatomy of the LV PPM can vary significantly, with antero-lateral and postero-medial PPM not always fully individualized on cardiac imaging^32^.

The aforementioned analysis allowed to measure the PPM mass, LV myocardial mass, LV EDV, and LV ESV, as well as the LV SV and LV EF. Only ED measurements were used to determine the PPM and LV myocardial masses. The LV SI was also computed as the ratio between the short- and long-axis LV dimensions, as described in the literature^62,63^.

RWT was measured from a basal short-axis slice, located immediately basal to the tips of the PPM, with all measurements taken at ED^61,64^. Wall thickness measurements, including the IVSd and LVPWd, were made perpendicular to the LV wall to ensure accuracy. LVEDd was also measured to compute the RWT using Eq. 1.

1 $${\rm{RWT}}=\,\frac{{\rm{IVSd}}+{\rm{LVPWd}}}{{\rm{LVEDd}}}$$

As part of the LV function assessment, GLS and GCS were analyzed. To calculate these global strain parameters, the strain module of the analysis software was used. Cine images were uploaded from SAX, 2CV, 3CV, and 4CV. The epicardium and endocardium were segmented at the ED and tracked throughout systole using a feature tracking algorithm to detect ventricular deformation, as described in Brandt et al.^65,66^.

Native T1 and T2 were postprocessed according to the latest consensus statements^53,61,67^. After visual assessment to detect artifacts and significant motion, quantitative analysis was conducted using a single region of interest manually drawn conservatively in the interventricular septum on the mid-cavity short-axis using the grayscale image.

The MAD was assessed by measuring the linear distance between the mitral leaflet insertion points on the septal and lateral sides of the annulus. This was performed on the long axis of the 2CV and 4CV at ED^68^.

### Statistical analysis

Statistical analysis was performed using GraphPad Prism for macOS 10.2.0. (GraphPad Software, Boston, United States) and RStudio (Posit Software PBC, Boston, The United States) with R version 4.2.1. As neither body mass nor body surface area (BSA) changed between pre- and post-flight, indexing key MRI variables to BSA was not deemed necessary. Continuous variables were compared using paired *t* tests after assessing for outlying values, normality (QQ plot, Shapiro–Wilk, and D’Agostino–Pearson tests), and asserting a high within-pair Pearson correlation coefficient to justify the use of a large-sample test considering the reduced sample size. Statistical test diagnostics were made on residuals to assess the reliability of statistical conclusions. For tissue mapping, two different MRI field strengths were used: 1.5 T and 3 T. Therefore, only data acquired from cosmonauts tested pre- and post-flight on the same MRI scanner were considered for analysis, thus reaching a sample size of seven instead of nine. These two independent subgroups (1.5 T and 3 T) were pooled together for statistical analysis, and the Wilcoxon matched-pairs signed-rank test (a non-parametric test) was chosen, because of the bimodal nature of this distribution, to compare the pre- and post-flight timepoints for native T1 and T2.

For all features, a reliability assessment was performed to evaluate the intra-rater reliability and inter-rater agreement. All acquisitions were analyzed and considered in these assessments. For this purpose, the ICCs were computed based on a two-way mixed-effects model using two raters by applying the *psych* library with the function *ICC()* in R. The measurements from the first rater were taken as the actual measurements used in the statistical tests^69^. Based on the 95% confidence interval of the ICC estimate, values < 0.5, between 0.5 and 0.75, between 0.75 and 0.9, and > 0.90 were considered indicative of poor, moderate, good, and excellent reliability, respectively^69^. Moreover, possible bias between raters was assessed by the Bland-Altman analysis. Correlation analysis was conducted by computing the Pearson correlation coefficient and simple linear regression. Correlation analysis using tissue mapping variables was conducted using the percentage of changes between pre- and post-flight, considering the previously mentioned pooled distribution.

Continuous variables are expressed as the mean ± standard deviation. All tests were two-tailed, and a *P* value < 0.05 was considered statistically significant. Considering this type of study with a limited number of cosmonauts, a *P* value < 0.1 was also considered to uncover possible trends. Cohen’s *d* effect size parameter was computed for all results considered significant or for uncovering possible trends using the *rstatix* library with the function *cohens_d()* in R.

## Data Availability

The datasets used and/or analyzed during the current study are available from the corresponding author on a reasonable request.
